# The effect of citrus flavonoid extract supplementation on anaerobic capacity in moderately trained athletes: a randomized controlled trial

**DOI:** 10.1186/s12970-020-00399-w

**Published:** 2021-01-06

**Authors:** Lieke E. van Iersel, Yala R. Stevens, Jose M. Conchillo, Freddy J. Troost

**Affiliations:** 1 BioActor BV, Gaetano Martinolaan 85, 6229 GS Maastricht, The Netherlands; 2 Division Gastroenterology-Hepatology, Department of Internal Medicine, School of Nutrition and Translational Research in Metabolism (NUTRIM), Maastricht University, P.O. Box 616, 6200 MD Maastricht, The Netherlands; 3 Department of Food Innovation and Health, Centre for Healthy Eating and Food Innovation, Maastricht University, 5911 BV Venlo, The Netherlands

**Keywords:** Antioxidant, Wingate anaerobic test, Anaerobic capacity, Hesperidin

## Abstract

**Background:**

Nutritional supplementation is commonly used by athletes to improve their exercise performance. Previous studies demonstrated that citrus flavonoid extract (CFE) supplementation may be an effective strategy to improve exercise performance in male athletes. Yet, no conclusive research has been performed to investigate the effect of chronic CFE supplementation on high-intensity exercise performance under anaerobic conditions. Therefore, the aim of the study was to assess whether CFE supplementation in daily dosages of 400 and 500 mg for a period of 4 and 8 weeks improves anaerobic exercise capacity.

**Methods:**

A randomized, double-blind, placebo controlled, parallel clinical study was conducted in 92 moderately trained healthy men and women. Subjects were randomized to receive 400 mg of CFE (*n* = 30), 500 mg of CFE (*n* = 31) or placebo (*n* = 31) daily, for 8 consecutive weeks. The Wingate anaerobic test was used to assess anaerobic exercise capacity and power output at baseline, after 4 weeks and after 8 weeks.

**Results:**

After 4 weeks supplementation, average power output significantly increased in the 400 mg group (Estimated difference [ED] = 38.2 W [18.0, 58.3]; *p* < 0.001; effect size [ES] = 0.27) and in the 500 mg group (ED = 21.2 W [0.91, 41.4]; *p* = 0.041; ES = 0.15) compared to placebo. The 5 s peak power output was also increased in the 400 mg group (ED = 53.6 [9.96, 97.2]; *p* = 0.017; ES = 0.25) after 4 weeks compared to placebo. After 8 weeks of supplementation, average power output was significantly improved in the group receiving 400 mg of CFE (ED = 31.6 [8.33, 54.8]; *p* = 0.008; ES = 0.22) compared to placebo.

**Conclusion:**

These results demonstrate that CFE supplementation improved anaerobic capacity and peak power during high intensity exercise in moderately trained individuals. Further research is needed to identify the underlying mechanisms that are affected by CFE supplementation.

**Trial registration:**

ClinicalTrials.gov (NCT03044444). Registered 7 February 2017

**Supplementary Information:**

The online version contains supplementary material available at 10.1186/s12970-020-00399-w.

## Introduction

Nutritional supplementation is commonly used by athletes to improve their exercise performance [1]. Supplementation with vitamins, antioxidants, creatine, proteins and carbohydrates aiming at enhancing exercise performance and muscle recovery has become of great interest in recent years, as these supplements may have beneficial effects on metabolic activity, strength and overall exercise performance [2–4]. During exercise, skeletal muscle mitochondria are of pivotal importance for the production of energy in the form of adenosine triphosphate (ATP), which is produced trough mitochondrial respiration. However, during the first 30 s of very high intensity-exercise, the phosphagen system is involved, producing immediate but limited energy under anaerobic conditions [5, 6].

It is considered that moderate exercise induces production of low concentrations of reactive oxygen species (ROS) in mitochondria, which subsequently act as a signal to stimulate the synthesis of enzymes that are involved in the adaptive response of the skeletal muscle, such as nitric oxide (NO) synthase and superoxide dismutase (SOD) [7–9]. However, the beneficial effects of low ROS levels will be lost when physical activity becomes strenuous [8]. During high intensity exercise, muscles rely at least in part on anaerobic metabolism. Anaerobic energy expenditure increases the production of fatigue associated products, mainly lactate and high levels of ROS, which contributes to impaired muscle functioning and oxidative damage as the body’s antioxidative scavenging capacity may be exceeded [2, 5, 6]. Subsequently, the oxidant/antioxidant balance becomes skewed in favor of free radical production, resulting in oxidative stress, structural cell damage and potentially also inflammation [8].

To prevent the negative consequences of excessive free radical accumulation during high intensity exercise, daily polyphenol supplementation can be an effective strategy to enhance muscle strength and improve muscle recovery. Polyphenols are compounds, which are naturally present in plants. They can be divided into different classes, based on their chemical structure, of which flavonoids are the largest group. Recent studies have shown anti-inflammatory and antioxidant effects of polyphenols which are present in fruits, such as blueberries, cherries, pomegranate and citrus fruit [10, 11]. In addition, a clinical study with polyphenol-rich extract supplementation showed an increased maximal power output, average power and total power output during high intensity exercise, without inducing more fatigue [2]. Furthermore, a translational study demonstrated that citrus flavonoid treatment improved vascular function by reducing circulating inflammatory biomarkers and stimulation of NO production [12]. It is suggested that increased NO production is associated with endothelium-dependent vasodilation in the arterial wall, causing a reduction in blood pressure. In relation with exercise physiology, blood flow to active muscles will be improved [11]. Hereby, fatigue-related metabolites are removed quickly and nutrient and oxygen delivery to active muscles is enhanced, improving tolerance to physical exercise and muscle recovery mechanisms [13].

A previous study with flavonoids from citrus fruit showed that repeated intake of 500 mg of a specific citrus flavonoid extract (CFE) improved aerobic exercise performance during a ten-minute bicycle trial on an ergometer. An increase in absolute power output and decrease in oxygen consumption/power ratio in trained male athletes was observed after 4 weeks of CFE treatment [10]. Furthermore, acute intake of a single dose of 500 mg CFE resulted in an increase in average power, maximum speed and total energy during the best sprint in an anaerobic cycling performance test, the Wingate Test [14]. However, it is unknown if chronic CFE supplementation also affects anaerobic exercise performance and whether such effects may also be achieved by lower daily supplementation dosages.

Therefore, the aim of the present clinical study was to investigate whether supplementation with CFE at dosages of 400 mg and 500 mg for a duration of 4 and 8 consecutive weeks improves anaerobic physical performance in moderately trained athletes during a Wingate Anaerobic Test (WAnT). It was hypothesized that CFE supplementation improves anaerobic exercise capacity in moderately trained individuals.

## Methods

The study was approved by the Medical Ethics Committee of Wageningen University, Wageningen, The Netherlands, and was conducted in full accordance with the principles of the Declaration of Helsinki of 1975 as amended in 2013 (Fortaleza, Brazil) and with the Dutch Regulations on Medical Research involving Human Subjects (1998). All participants gave written informed consent before participation. The study was performed at Topsport Limburg High Performance Center, Sittard, The Netherlands between April 2017 and April 2018 and has been registered at clinicaltrials.gov (NCT03044444).

### Subjects

Healthy, non-smoking, moderately trained volunteers, both male and female, aged 18–35 years and Body Mass Index (BMI) between 18 and 30 kg/m^2^ were recruited through advertisements in local media and through partnership with Topsport Limburg High Performance Center, Sittard, The Netherlands. Participants were amateur athletes in resistance or interval sports, engaging in moderate physical activity for a minimum of 4 h a week. Exclusion criteria were as follows: the use of medication and ergogenic supplements (e.g. creatine or anabolic steroids) that may interfere with study outcomes; intake of antioxidant/vitamin supplements; cardiovascular complications; allergy to study product or placebo; intake of dietary products containing citrus flavonoids or metabolites; and abuse of alcohol (> 20 alcoholic units/wk) or recreational drugs.

### Design and protocol

**Fig. 1:**
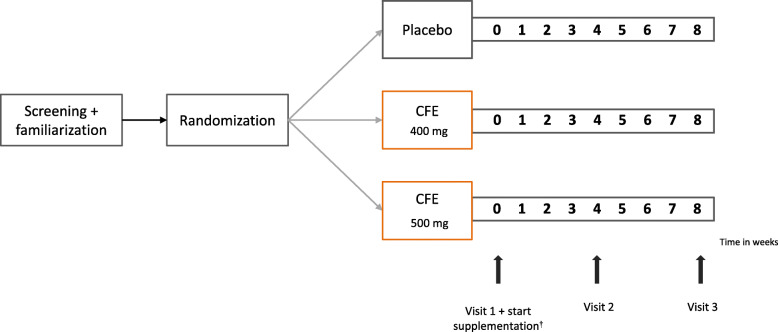
Overview of the study design. CFE: citrus flavonoid extract. ^†^ Supplementation started after baseline measurements were completed

The study was designed as a randomized, double-blind, placebo-controlled parallel study to test the effects of a daily dosage of 400 mg CFE supplementation or 500 mg CFE supplementation versus placebo over a period of 4 and 8 consecutive weeks (Fig. 1). Previous human intervention studies focusing on exercise performance or other relevant outcomes such as endothelial function have been performed using a citrus flavonoid (extract) supplementation dosage of 500 mg [10, 12, 14, 15]. However, so far, information about the effectiveness at lower dosages is limited. The present study aimed to test whether the same putative performance outcomes over the 8-weeks supplementation period can be achieved with a lower dosage of CFE supplementation, as this would benefit future applications. To the best of our knowledge, this is the first human study investigating the effects of long-term CFE supplementation on anaerobic physical performance at a daily dose of 500 mg, as well a 20% lower daily dose of 400 mg.

Participants were randomly assigned to one of the following three intervention groups: CFE 400 mg, CFE 500 mg or placebo. A randomization list was generated by an independent person using a randomization program (http://randomizer.org). The study consisted of a familiarization test, a baseline test (visit 1), a test day after 4 weeks of intervention (visit 2) and a test day after 8 weeks of intervention (visit 3). During the intervention period, participants ingested CFE 400 mg, CFE 500 mg or placebo supplements q.d. (once per day). Subjects were instructed to abstain from high intensity exercise and consumption of caffeine and alcohol 24 h prior to each test day. During the entire study period, participants were instructed to refrain from consuming foods containing high levels of citrus flavonoids (i.e. lemons, oranges and grapefruit) and to maintain their habitual dietary intake and weekly training schedule. At the start of each test visit, subjects handed in a 3-day dietary record.

The familiarization test was performed in order to get familiar with the testing procedures and exercise protocol. At the familiarization test, subjects performed the WAnT, preceded by a standardized warm-up protocol. The test protocol was performed identically on each test day.

### Wingate anaerobic test

The WAnT was performed on a Wattbike Performance Monitor (WPM) (Wattbike Ltd., Nottingham, UK). Before warm-up, the subjects were provided with a Garmin HRM1G-Heart Rate Monitor. During the familiarization test the optimal settings for saddle height, saddle position and steer height and position were determined and recorded. The resistance level was calculated based on gender and bodyweight by the WPM [16]. The resistance was set at 0.095 kg/kg bodyweight for men and 0.086 kg/kg bodyweight for women [17]. The warm-up consisted of 14 min of cycling at ±100 W. After 10, 11 and 12 min, the subjects were requested to sprint all-out for 3 s. Subsequently, subjects rested for exactly 1 min and after a 5 s countdown, the subjects started to sprint for 30 consecutive seconds, conform to the standard WAnT protocol [18]. They were instructed to generate the maximal total power output (W) possible over the time course of 30 s. After 30 s, the resistance was set back and the subject cycled on slow pace for 3 min after which the test was finished. The WPM was interfaced with Wattbike Expert Ver. 2.60.20 (Wattbike Ltd., Nottingham, UK), which assessed average power (AP) and 5 s peak power (5sPP) to determine anaerobic capacity and anaerobic peak power. In addition, Time to Peak in seconds was measured and heart rate (HR) in beats per minute (bpm) was monitored continuously over the 30 s sprint trial, to assess the maximum HR.

### Study product

The test product is a *Citrus sinensis* extract containing 90% hesperetin-7-O-rutinoside of which > 75% is comprised of the 2S enantiomer (WATTS’UP®, BioActor BV, Maastricht, the Netherlands). Microcrystalline cellulose (Microz, Geleen, the Netherlands) was used as placebo. The study products were formulated into capsules. The placebo and CFE500 contained 250 mg study product per capsule, the CFE400 capsules contained 200 mg study product and 50 mg microcrystalline cellulose. Furthermore, the study products were produced to be identical in flavor and appearance. The subjects were instructed to orally ingest two capsules with 200 mL water each morning before consuming breakfast. The total amount of hesperidin in the CFE400 and CFE500 supplements contained the equivalent of approximately 0.7–0.9 L of commercially available sweet orange juice [19].

### Dietary intake

Dietary intake was assessed by 3-day dietary records as described previously [20]. Briefly, participants were asked to record the intake of 2 week days and one weekend day before each test day, based on standard household units.

### Statistical analysis

Statistical analysis was performed using IBM SPSS Statistics for Windows (version 25.0, IBM Corporation, Armonk, NY, USA). The sample size was determined with a significance level of α = 0.05 and a power of 90%. Based on previous work [10], a sample size of 78 participants was required to reach sufficient statistical power. Data are presented as observed mean ± standard deviation (SD) for numerical variables and numbers for categorical variables. Differences in outcomes between the intervention groups (i.e. CFE400 and CFE500) and the placebo were assessed by unstructured linear mixed model analyses. Intervention, time and intervention x time were included as fixed factors. This model accounts for the correlation between repeated measures and missing data. Intention to treat analyses were performed for all outcomes. The estimated mean difference in change scores from baseline between the intervention groups and the placebo [95% confidence interval] obtained from this model are reported, matching with the intention to treat analysis and taking missing data into account. The effect size (ES) was determined by dividing the estimated mean difference by the square root of the estimated baseline residual variance obtained from the model. Within-group differences were not tested. Level of significance was set on *p* < 0.05.

## Results

### Study subjects

**Fig. 2:**
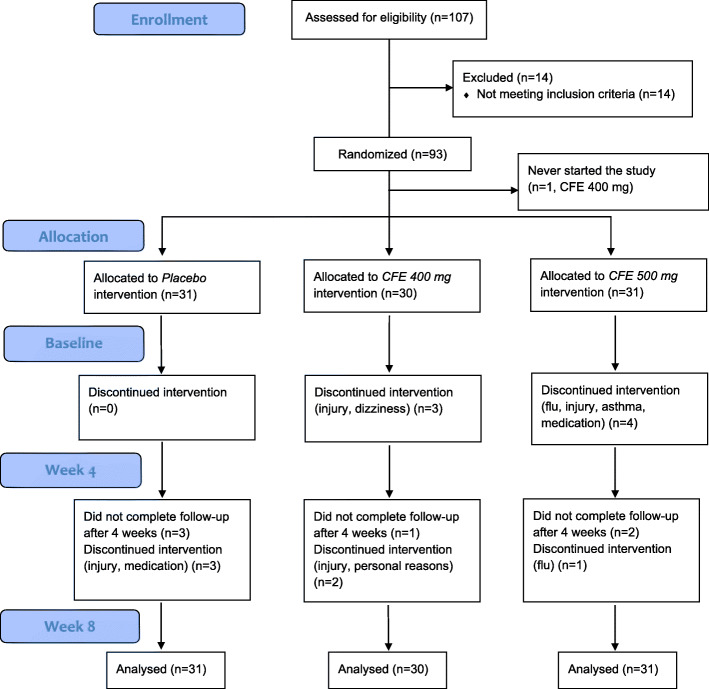
CONSORT (Consolidated Standards of Reporting Trials) flowchart of the study

In total, 93 healthy subjects were enrolled in the study. Seventy-nine subjects completed the exercise test (WAnT) after 4 weeks supplementation as well as after 8 weeks supplementation. One subject, allocated to the CFE400 group, did not start the study due to private circumstances Thirteen subjects did not complete the second test day after 4 weeks of supplementation due to injuries or illness not related to the study. In total, 79 subjects completed the third test day after 8 weeks of supplementation. (Fig. 2). Baseline characteristics of the study population are shown in Table 1.

**Table 1:** Baseline participant characteristics

	Total (*N* = 92)	Placebo (*N* = 31)	CFE400 (*N* = 30)	CFE500 (*N* = 31)	*P*-value
Male/Female (*N*)	52/40	20/11	15/15	17/14	0.506
Age (years)	24 ± 5	24 ± 5	24 ± 5	23 ± 4	0.361
Weight (kg)	70.8 ± 11.0	71.9 ± 11.5	68.7 ± 11.7	71.7 ± 9.8	0.445
Height (m)	1.77 ± 0.1	1.77 ± 0.1	1.76 ± 0.1	1.79 ± 0.1	0.275
BMI (kg/m^2^)	22.4 ± 2.2	23.0 ± 2.0	22.2 ± 2.3	22.2 ± 2.2	0.262
Exercise time (h)	7.94 ± 4.15	8.11 ± 4.6	7.63 ± 3.6	8.07 ± 4.4	0.885
Average Power (W)	543 ± 143	547 ± 141	511 ± 134	571 ± 149	0.259
5 s Peak Power (W)	721 ± 215	726 ± 228	675 ± 200	762 ± 214	0.286
Time to peak (s)	1.73 ± 2.2	1.57 ± 3.0	2.37 ± 1.9	1.28 ± 1.1	0.131
Heart Rate Maximum (BPM)	173 ± 11	171 ± 9	173 ± 14	173 ± 10	0.699

Values are presented as mean ± SD. Age, Weight, Height, BMI, Exercise time, Average Power, 5 s Peak Power, Time to peak and Heart Rate Maximum were compared between intervention groups with the use of a one-way analysis of variance (ANOVA). Gender was compared between intervention groups with the use of a Pearson’s chi-square test. BMI for all participants was between 19.40 and 29.10 kg/m^2^. *CFE* Citrus Flavonoid Extract. *BMI* Body Mass Index

### Average power output after 4 weeks

**Fig. 3:**
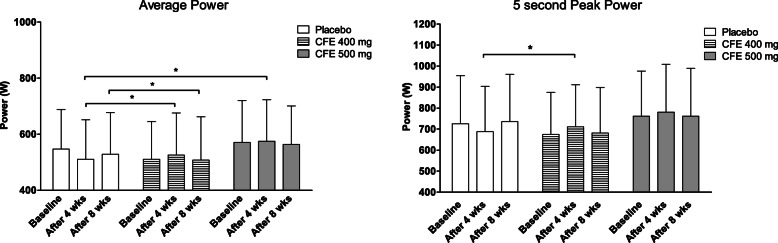
Performance outcomes at baseline, after 4 weeks and after 8 weeks of supplementation. Values are presented as observed mean ± SD. Differences between the intervention groups and placebo group were compared with an unstructured linear mixed model with correction for baseline values. * *p* < 0.05. CFE: Citrus Flavonoid Extract

No significant differences in body weight were observed over time in the placebo and the two treatment groups. Therefore, the performance outcomes were not corrected for body weight. The results of the average power output from the participants supplemented with CFE and placebo, before and after 4 weeks intervention are summarized in Table 2 and Fig. 3. When comparing the differences in AP output between the CFE400 group and placebo, a significant effect (*p* < 0.001; ES = 0.27) was found after 4 weeks of treatment. The CFE500 supplementation also resulted in a significant difference in AP output versus placebo (*p* = 0.041; ES = 0.15).

**Table 2:** Performance outcomes at baseline and after 4 weeks of supplementation, with corresponding *p*-values

Variable	Placebo (*N* = 31)		CFE400 (*N* = 30)		CFE500 (*N* = 31)		Estimated difference [95% CI]1	*Effect size1*	*P1*	Estimate [95% CI]2	*Effect size2*	*P2*
	Baseline	4 weeks^†^	Baseline	4 weeks^¥^	Baseline	4 weeks^§^						
**Weight (kg)**	71.9 ± 11.5	70.9 ± 11.8	68.7 ± 11.7	67.5 ± 11.7	71.7 ± 9.8	72.6 ± 9.3	−0.01 [− 0.80, − 0.78]	−0.001	*0.984*	−0.19 [− 0.98, 0.59]	−0.02	*0.621*
**Average Power (W)**	547 ± 141	511 ± 140	511 ± 134	526 ± 150	571 ± 149	575 ± 148	38.2 [18.0, 58.3]	0.27	< 0.001	21.2 [0.91, 41.4]	0.15	*0.041*
**5 s Peak Power (W)**	726 ± 228	688 ± 216	675 ± 200	711 ± 211	762 ± 214	781 ± 227	53.6 [9.96, 97.2]	0.25	*0.017*	28.7 [− 15.2, 72.5]	0.13	*0.197*
**Time to peak (s)**	1.57 ± 3.0	1.48 ± 1.4	2.37 ± 1.9	1.19 ± 0.8	1.28 ± 1.1	0.98 ± 0.8	−1.08 [−2.21, 0.05]	−0.50	*0.060*	−0.20 [− 1.32, 0.93]	−0.09	*0.730*
**Heart Rate Maximum (BPM)**	171 ± 9	168 ± 11	173 ± 14	175 ± 13	173 ± 10	171 ± 11	3.82 [− 1.08, 8.71]	0.34	*0.124*	0.06 [−4.75, 4.87]	0.01	*0.980*

Values are presented as observed mean ± SD. Differences between the intervention groups and placebo group were compared with an unstructured linear mixed model with correction for baseline values. Estimated difference 1: Estimated difference in change scores from baseline between CFE400 and placebo obtained with the mixed model. Estimated difference 2: Estimated difference in change scores from baseline between CFE500 and placebo obtained with the mixed model. *CI* Confidence interval. Effect size1: effect size CFE400 vs. placebo. Effect size2: effect size CFE500 vs and placebo. P1: *P*-value between CFE400 and placebo. P2: *P*-value between CFE500 and placebo. *CFE* Citrus Flavonoid Extract. † Data from all 31 participants were included in the analysis, 28 participants actually completed the measurements after 4 weeks. ¥ Data from all 30 participants were included in the analysis, 26 participants actually completed the measurements after 4 weeks. § Data from all 31 participants were included in the analysis, 25 participants actually completed the measurements after 4 weeks

### Peak power output after 4 weeks

The results of the peak power output from the participants supplemented with CFE and placebo, before and after 4 weeks intervention are summarized in Table 2 and Fig. 3. When comparing the differences in 5sPP between baseline and after 4 weeks of supplementation, a significant effect between the CFE400 group and placebo was found (*p* = 0.017; ES = 0.25), but no significant difference in 5sPP was found in the intervention group receiving CFE500 versus placebo (*p* = 0.197; ES = 0.13).

Additionally, a slight decrease in Time to peak was observed after 4 weeks in the CFE400 group compared to the placebo (*p* = 0.060; ES = -0.50). No changes in HR were observed between the groups after 4 weeks of supplementation.

### Average power output and peak power output after 8 weeks

AP output improved significantly in the CFE400 group compared to placebo after 8 weeks of supplementation (*p* = 0.008; ES = 0.22; Table 3; Fig. 3). Supplementation with CFE500 for 8 weeks did not result in significant differences in AP output versus placebo (*p* = 0.471; ES = 0.06). No significant changes in 5sPP were found in the CFE400 group nor the CFE500 group versus placebo after 8 weeks of intervention (Table 3; Fig. 3).

**Table 3:** Performance outcomes at baseline and after 8 weeks of supplementation, with corresponding *p*-values

Variable	Placebo (*N* = 31)		CFE400 (*N* = 30)		CFE500 (*N* = 31)		Estimated difference [95% CI]1	*Effect size1*	*P1*	Estimated difference [95% CI]2	*Effect size2*	*P2*
	Baseline	8 weeks^†^	Baseline	8 weeks^¥^	Baseline	8 weeks^§^						
**Weight (kg)**	71.9 ± 11.5	72.6 ± 13	68.7 ± 11.7	67.6 ± 11.9	71.7 ± 9.8	73.1 ± 9.4	−0.26 [− 1.21, 0.69]	− 0.02	*0.591*	− 0.39 [− 1.33, 0.66]	−0.04	*0.411*
**Average Power (W)**	547 ± 141	528 ± 149	511 ± 134	508 ± 154	571 ± 149	564 ± 137	31.6 [8.33, 54.8]	0.22	*0.008*	8.43 [−14.7, 31.6]	0.06	*0.471*
**5 s Peak Power (W)**	726 ± 228	736 ± 255	675 ± 200	682 ± 216	762 ± 214	762 ± 228	29.4 [− 18.2, 77.1]	0.14	*0.222*	−11.5 [− 58.8, 35.9]	−0.05	*0.631*
**Time to peak (s)**	1.57 ± 3.0	0.91 ± 0.7	2.37 ± 1.9	1.17 ± 1.8	1.28 ± 1.1	1.8 ± 4.8	−0.15 [− 2.16, 1.86]	−0.07	*0.882*	1.07 [−0.92, 3.06]	0.49	*0.290*
**Heart Rate Maximum (BPM)**	171 ± 9	166 ± 14	173 ± 14	173 ± 18	173 ± 10	169.7 ± 13	2.30 [− 4.10, 8.69]	0.20	*0.477*	− 0.17 [− 6.58, 6,25]	−0.01	*0.958*

Values are presented as observed mean ± SD. Differences between the intervention groups and placebo group were compared with an unstructured linear mixed model with correction for baseline values. Estimated difference 1: Estimated difference in change scores from baseline between CFE400 and placebo obtained with the mixed model. Estimated difference 2: Estimated difference in change scores from baseline between CFE500 and placebo obtained with the mixed model. *CI* Conficence interval. Effect size1: effect size CFE400 vs. placebo. Effect size2: effect size CFE500 vs and placebo. P1: *P*-value between CFE400 and placebo, P2: *P*-value between CFE500 and placebo. *CFE* Citrus Flavonoid Extract. † Data from all 31 participants were included in the analysis, 28 participants actually completed the measurements after 8 weeks. ¥ Data from all 30 participants were included in the analysis, 25 participants actually completed the measurements after 8 weeks. § Data from all 31 participants were included in the analysis, 26 participants actually completed the measurements after 8 weeks.

### Dietary intake

The intake of energy and macronutrients of the participants was assessed during the intervention period (Table s1). No significant differences in dietary intake (not in total energy intake, nor in fat-, protein-, and carbohydrate intake) between the intervention groups were observed throughout the study period.

## Discussion

This study determined the effects of 8-weeks CFE supplementation in different dosages on exercise performance under anaerobic conditions in moderately trained individuals. Both CFE dosages (400 and 500 mg q.d., respectively) significantly increased anaerobic exercise performance as assessed by average power output during the Wingate test after 4 weeks, compared to placebo. In the CFE400 group, but not in the CFE500 group, this was accompanied by a significant increase in peak power output, as indicated by the 5sPP. After 8 weeks of supplementation, the anaerobic exercise performance was still increased in the CFE400 group compared to placebo. No differences were observed after 8 weeks of CFE500 supplementation.

The effect of polyphenols or antioxidants on endurance exercise performance has been investigated in previous studies, demonstrating that polyphenol supplementation can improve performance outcomes in study populations ranging from healthy but untrained individuals to trained athletes [10, 21–24]. However, research into the effects of polyphenol supplementation on anaerobic capacity is less extensive. In the present study, we showed that daily supplementation with CFE400 increased anaerobic capacity in moderately trained individuals. After 4 weeks of CFE supplementation, AP was also significantly improved in the CFE500 group and the 5sPP output increased in the CFE400 group, both compared to placebo. This increase did not result in a concomitant increase in maximum heart rate during the exercise.

The outcomes of 4 weeks CFE treatment are in line with those from previous studies. In male amateur cyclists, a single dose of CFE administered 5 h before the exercise session increased average power by 2.3%, maximum speed by 3.2% and total energy by 2.6% when data of the best of 4 sprints was considered. No significant improvements of this acute dose were observed for the average results [14]. In a study investigating supplementation of a polyphenol-rich extract, peak power was shown to be increased by 3.7% and average power by 5%, without a concomitant increase in maximum HR [2]. That study demonstrated that polyphenol supplementation did not affect heart workload despite the increased power outputs, probably due to a decreased blood pulse pressure, which is considered to be a biomarker for heart workload [2, 25]. Despite the fact that blood pulse pressure was not measured in the current study, the same results in HR were observed. It might be possible that CFE supplementation decreased intravenous resistance as some studies showed that polyphenol supplementation is related to an increased endothelial NO production [12, 13]. In the present study, a higher dosage of CFE did not result in better performance output, as the CFE 400 overall showed a stronger improvement in exercise performance outcomes than the CFE500 compared to placebo both after 4 and after 8 weeks of supplementation. This finding was surprising, as we were expecting no difference in performance. As such, it is tempting to speculate on the possible explanation for these findings. One potential explanation might be that the higher dosage may have reduced the ROS concentrations within the skeletal muscle to a larger extent, resulting in concentrations that were less optimal for muscle contractility [26].

We included healthy, moderately trained male and female subjects. Based on the result of the current study, strategic supplementation with CFE might be of interest for recreational athletes competing in sports that have a large anaerobic component, such as sprinters and track cyclists. Although the reported effects are small, these observed differences may be of high relevance and impact, if these results of this study in recreational athletes can be extrapolated to highly trained sports professionals. In elite athletes, small differences may determine winning or losing a competition. Further research is needed to provide more insight into the effects of CFE supplementation in elite athletes, to substantiate CFE use as a nutritional ergogenic aid.

Although CFE supplementation increased anaerobic exercise performance in moderately trained subjects after supplementation with CFE, the underlying mechanism remains to be elucidated. A putative mechanism of action may be that CFE increases oxygen delivery to the muscles by upregulating NO with a subsequent vasodilation and increased blood flow response, thereby also increasing the removal of waste products, such as lactate. For instance, hesperetin, a metabolite from the citrus flavonoid hesperidin, has been associated with an increased NO release from endothelial cells and hesperidin supplementation in human subjects has been associated with improved endothelial function [12, 15, 27]. Furthermore, previous studies have demonstrated that hesperidin reduces oxidative stress levels by scavenging ROS and improving antioxidative capacity, which is especially beneficial for high intensity anaerobic exercise as anaerobic functioning leads to increased production of muscle fatigue associated end-products such as lactate and high ROS levels [28–30]. Another potential mechanism of action might involve modulation of mitochondrial metabolism. It has been demonstrated that treatment with hesperetin increases intracellular ATP by 33% and mitochondrial spare capacity (i.e. the difference between maximum and basal respiratory capacity) by 25% in myotubes [31], which could result in reduced oxidative stress levels and more ATP availability during high intensity exercise.

Some potential limitation of the current study design should be mentioned. Before the start of the study, a familiarization test was conducted to minimize learning effects of the WAnT exercise protocol. Nevertheless, a learning effect on the WAnT outcome cannot entirely be excluded, although the placebo arm did not show such learning effects over the consecutive tests, and the randomized design of the study will have minimized potential confounding of a potential learning effect. Another limitation of this study is that participants were not randomized based on training level, mode of training, intensity of training or frequency of training. All participants were moderately trained, but some variation in the aforementioned factors may have been present, resulting in high standard deviations. However, we did not observe significant differences in training hours per week or any of the baseline performance outcomes between groups. Furthermore, this study did not investigate the mechanisms which underlie the observed differences in anaerobic exercise performance. Blood collection and muscle biopsies would have given more insight into the effect of hesperidin on oxidative stress levels, inflammation levels and mitochondrial function [31]. These tests were not included in the current study in order to limit the test burden for participants.

## Conclusions

This study shows that daily intake of CFE, a natural flavonoid containing supplement, resulted in increased anaerobic capacity and peak power output during high intensity exercise in moderately trained individuals without affecting the maximum heart rate. Future research needs to be performed to identify the underlying mechanisms that are affected by CFE supplementation.

## Supplementary Information

**Additional file 1: Table S1.** Dietary intake at baseline, after 4 weeks and after 8 weeks of supplementation, with corresponding *p*-values.

## Data Availability

The datasets used and/or analyzed during the current study are available from the corresponding author on reasonable request.

## Abbreviations

5sPP: 5 s Peak power
AP: Average power
ATP: Adenosine triphosphate
BMI: Body mass-index
Bpm: Beats per minute
CFE: Citrus flavonoid extract
HR: Heart rate
NO: Nitric oxide
ROS: Reactive oxygen species
SD: Standard deviation
SOD: Superoxide dismutase
WAnT: Wingate anaerobic test
WPM: Wattbike performance monitor
