# Supplementary material for: The effect of citrus flavonoid extract supplementation on anaerobic capacity in moderately trained athletes: a randomized controlled trial
Source: J Int Soc Sports Nutr. 2021 Jan 6;18:2. doi: 10.1186/s12970-020-00399-w (PMC7789554; doi:10.1186/s12970-020-00399-w)
Supplement: Supplementary file 1 — Additional file 1: Table S1. Dietary intake at baseline, after 4 weeks and after 8 weeks of supplementation, with corresponding p-values. [file 12970_2020_399_MOESM1_ESM.pdf]

Supplementary material

Table s1. Dietary intake at baseline, after 4 weeks and after 8 weeks of supplementation, with corresponding p-values.

		Placebo	CFE400	CFE500	*P1*	*P2*
**Energy (kcal)**	**Baseline**	2359 ± 788	2262 ± 569	2592 ± 762		
	**4 weeks**	2404 ± 706	2366 ± 614	2462 ± 692	0.596	0.293
	**8 weeks**	2460 ± 993	2126 ± 694	2615 ± 697	0.245	0.592
**Fat (g)**	**Baseline**	87.7 ± 36.7	81.4 ± 24.1	91.2 ± 30.2		
	**4 weeks**	92.3 ± 30.6	90.6 ± 23.8	91.9 ± 28.3	0.599	0.459
	**8 weeks**	89.0 ± 41.5	72.3 ± 20.3	98.2 ± 29.2	0.138	0.723
**Protein (g)**	**Baseline**	106.6 ± 54.7	96.6 ± 24.0	115.8 ± 48.0		
	**4 weeks**	103.3 ± 39.2	99.0 ± 34.1	107.2 ± 47.4	0.370	0.777
	**8 weeks**	108.8 ± 49.3	89.0 ± 35.0	115.0 ± 44.2	0.326	0.974
**Carbohydrates (g)**	**Baseline**	263.4 ± 77.6	257.0 ± 85.0	298.5 ± 98.0		
	**4 weeks**	266.0 ± 93.9	255.1 ± 100.0	280.7 ± 87.4	0.875	0.520
	**8 weeks**	280.7 ± 123.7	256.2 ± 112.3	295.6 ± 98.4	0.756	0.452

Values are expressed as observed mean ± SD. Differences between the intervention groups and placebo group were compared with an unstructured linear mixed model with correction for baseline values. P1: P-value between CFE400 and placebo. P2: P-value between CFE500 and placebo.
